# The Dual Roles of Circular RNAs in Breast Cancer Distant Metastasis and Their Clinical Applications

**DOI:** 10.7150/jca.111680

**Published:** 2025-07-11

**Authors:** Qing Bao, Huan Zhang, Pangzhou Chen, Song Wu, Yutian Zou, Huan Wang, Jingna Cao, Wen Zhou, Ziyun Guan, Hailin Tang

**Affiliations:** 1 State Key Laboratory of Oncology in South China, Guangdong Provincial Clinical Research Center for Cancer, Sun Yat-sen University Cancer Center, Guangzhou, 510060, China.; 2 Guangzhou Kangda Vocational Technical College, Guangzhou, 510555, China.; 3 The Sixth Affiliated Hospital, School of Medicine, South China University of Technology, Foshan, China.

**Keywords:** Circular RNAs (circRNAs), Breast cancer metastasis, Epithelial‒mesenchymal transition (EMT)

## Abstract

Circular RNAs (circRNAs) are emerging as crucial regulators of the progression and metastasis of breast cancer. This review examines the dual roles of circRNAs, which act as both oncogenes and tumor suppressors, in breast cancer metastasis. CircRNAs play crucial roles in processes such as the epithelial‒mesenchymal transition (EMT), angiogenesis, immune evasion, and metabolic adaptation, facilitating cancer cell invasion, survival, and colonization in distant organs such as the brain, liver, bone, and lungs. Pro-metastatic circRNAs, such as circKIF4A and circBACH1, promote metastasis by modulating signaling pathways such as the STAT3 and PI3K/AKT pathways, whereas tumor-suppressive circRNAs, including circFOXO3 and circNFIB, inhibit metastatic progression through mechanisms such as VEGF downregulation and the suppression of arachidonic acid metabolism. Although circRNAs hold promise as biomarkers and therapeutic targets, their clinical application is impeded by challenges such as targeted delivery, off-target effects, and context-dependent roles. This review highlights the current understanding of circRNA-mediated regulation of breast cancer metastasis and emphasizes future directions, including the integration of multiomics technologies and advanced delivery systems, to increase the diagnostic and therapeutic utility of circRNAs.

## Introduction

Breast cancer is the most common malignancy in women worldwide, accounting for more than 11.6% of all cancer diagnoses 1. Metastasis is frequently linked to both a high incidence and poor prognosis 2. Breast cancer can be classified into several molecular subtypes, including hormone receptor-positive (ER+/PR+), human epidermal growth factor receptor 2-positive (HER2+), and triple-negative breast cancer (TNBC). TNBC is considered the most challenging subtype due to its aggressiveness and high risk of metastasis 3, 4. The World Health Organization (WHO) reports an increasing global incidence of breast cancer, with treatment challenges primarily involving metastasis and drug resistance 5. In breast cancer, distant metastasis arises when tumor cells disseminate from the primary site to other organs or tissues via the blood or lymphatic system. This phenomenon is often linked to a more aggressive tumor type and notably decreases patient survival rates 6. Metastasis involves biological mechanisms such as the epithelial‒mesenchymal transition (EMT), angiogenesis, and extracellular matrix (ECM) degradation 7, 8. Breast cancer frequently metastasizes to the bone, liver, lung, and brain 9, 10. Bone metastasis typically manifests as osteolytic lesions, which may result in pathological fractures and severe pain 11; liver metastasis is often associated with tumor invasiveness and high blood flow in the organ 12; and brain metastasis involves the breakdown of the blood‒brain barrier and generally has a poor prognosis 13.

The mechanisms of distant breast cancer metastasis involve multiple signaling pathways and molecular regulation, including the EMT, ECM degradation, angiogenesis, and immune evasion 7. CircRNAs, a new form of noncoding RNA, are essential for regulating distant metastasis processes in breast cancer. CircRNAs influence gene expression by sequestering specific microRNAs (miRNAs) or interacting with RNA-binding proteins (RBPs) to affect cellular behavior 14-17. Circular RNAs (circRNAs) are stable, covalently closed-loop RNA molecules formed via back-splicing and have unique biological functions 18. The main mechanisms involve acting as competing endogenous RNAs (ceRNAs) to modulate miRNA activity, interacting with RNA-binding proteins (RBPs), and directly encoding proteins 19-21. CircRNAs play roles in regulating cell proliferation, migration, invasion, angiogenesis, and drug resistance 22. In breast cancer, aberrant circRNA expression is significantly associated with tumor initiation, progression, and metastasis 23, 24.

In this study, we reviewed the latest advancements in the field and found that circRNAs play a key role in modulating the aggressiveness of breast cancer cells, as well as influencing distant metastasis and therapeutic resistance in breast cancer patients. Additionally, we identified that circRNAs regulate breast tumor angiogenesis, further contributing to cancer metastasis. Furthermore, through an extensive examination of numerous studies, we explored whether and how circRNAs could facilitate a diagnosis and prognostic predictions, as well as enhance the specificity of breast cancer treatment by serving as biomarkers. Many investigations have elucidated the influence of circRNAs on breast cancer metastasis through diverse signaling pathways, which constitutes a key aspect of this review.

## Biological Characteristics of CircRNAs in Breast Cancer

Circular RNAs (circRNAs) are noncoding RNAs with a covalently closed loop structure lacking a 5' cap and a 3' poly(A) tail 25, 26. Back-splicing, a noncanonical splicing process, is the primary mechanism of circRNA formation, setting them apart from linear RNAs. Most circRNAs are classified into three types based on their sequence origins: ecircRNAs, ciRNAs, and EIciRNAs 27. Research has shown that in breast cancer, many circRNAs contain known RBP binding sites that direct their circularization. These examples include circKIF4A, which is expressed from exon regions that can promote tumor development through its interactions with important signaling pathways 5, 28.

The closed-loop structure of circRNAs renders them highly stable by resisting exonuclease degradation. This stability enables circRNAs to accumulate in cells and bodily fluids, thus becoming attractive biomarkers for diseases such as breast cancer. In addition, circRNAs exhibit a high degree of conservation across species, which points toward their evolutionary importance 29. For example, circCDR1as is conserved across mammalian species and has been implicated in regulating oncogenic processes 23. The stability and conservation of circRNAs, such as circPVT1, are guaranteed in breast cancer tissues, leading to robust expression and the ability to serve as reliable diagnostic and prognostic markers.

Most likely, the best-described function of circRNAs that has been studied to date is acting as ceRNAs, which sponge miRNAs to regulate target genes at the downstream level. In ER-positive breast cancer, circPVT1 functions as a miR-181a-2-3p sponge, stabilizing the ESR1 mRNA and increasing estrogen receptor α (ERα) expression 30. Thus, this mechanism links circPVT1 to the proliferation of cells and drug resistance in breast cancer.

Some circRNAs, such as EIciRNAs, localize to the nucleus and interact with the transcriptional machinery. For example, circPAIP2 enhances the transcription of its parental gene through interactions with RNA polymerase II 31. In breast cancer, similar nuclear circRNAs may contribute to aberrant gene regulation, facilitating oncogenesis and metastasis. CircFOXO3 interacts with proteins such as p21 and CDK2 to regulate cell cycle progression and inhibit tumor growth 32.

**Figure 1:**
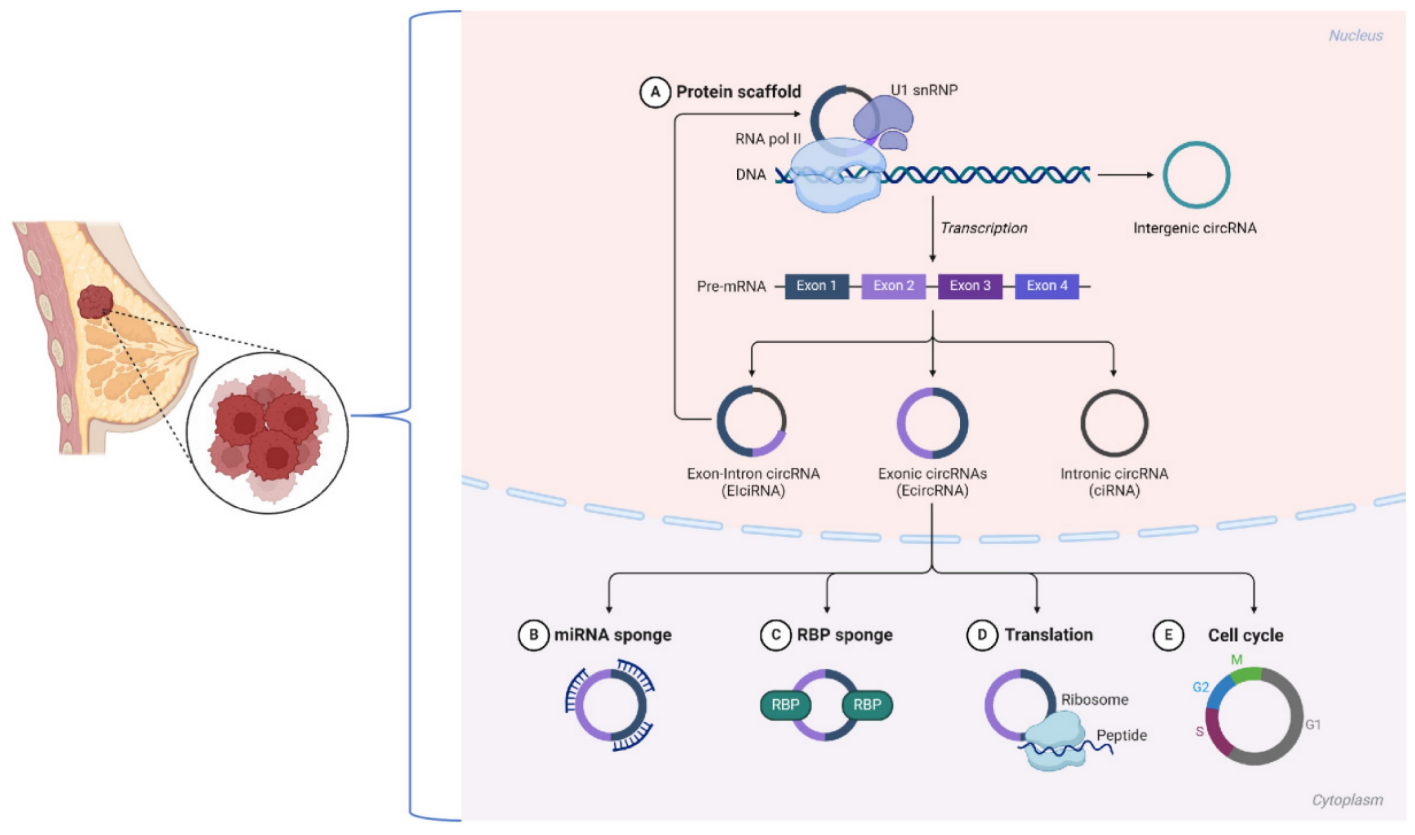
Biological mechanisms of circRNAs in breast cancer.

These mechanisms underscore the diverse functions of circRNAs in breast cancer biology. Recent advancements, including the use of CRISPR-Cas13d-based screening tools 33, have facilitated the identification of functional circRNAs, revealing their critical involvement in tumor progression, immune evasion, and therapy resistance. These findings underscore the potential of targeting circRNAs for therapeutic intervention in breast cancer (Figure 1).

## Circular RNAs that Enhance Breast Cancer Cell Invasiveness

### Circular RNAs that Promote Breast Cancer Cell Invasion and Migration

Recent research has indicated that circRNAs significantly increase breast cancer cell invasion and migration (Table 1). A study revealed that circKIF4A is overexpressed in TNBC, where it competitively binds to miR-637, modulating the STAT3 signaling pathway to promote brain metastasis and cell migration 3.

CircEZH2 is notably overexpressed in breast cancer patients with liver metastasis. The overexpression of circEZH2 significantly increases breast cancer cell viability and invasiveness. Conversely, when the expression of circEZH2 is knocked down, the resulting effects are precisely the opposite of those observed under overexpression conditions 34. The expression of circROBO1 is notably increased in breast cancer patients with liver metastases. Silencing circROBO1 significantly inhibits breast cancer cell proliferation, migration, and invasion. Conversely, its overexpression has the opposite effect, promoting these processes 35.

Studies using *in vitro* and *in vivo* models have shown that increased CircXPO6 expression promotes tumor progression and metastasis. CircXPO6 interacts with c-Myc to inhibit its ubiquitination and degradation by speckle-type POZ protein (SPOP), enhancing breast cancer cell migration and invasion 36. CircRAD18, a circRNA that is markedly upregulated in TNBC, has been identified in previous studies as a key regulator of breast cancer progression. By acting as a molecular sponge for miR-208a and miR-3164, it alleviates the inhibitory effects of these miRNAs on the IGF1 and FGF2 target genes, thus facilitating cell proliferation, increasing migration, and preventing apoptosis 37. Studies have indicated that circEPSTI1, a circRNA whose expression is markedly elevated in TNBC, acts as a miRNA sponge by interacting with miR-4753 and miR-6809. Through this interaction, it regulates BCL11A expression, thereby influencing TNBC cell proliferation and apoptosis 38.

In TNBC, overexpressed CircPLK1 can act as a ceRNA to regulate cell growth and invasion through the CircPLK1‒miR-296‒5p‒PLK1 axis 39. CircGFRA1, which is significantly upregulated in TNBC, functions as a ceRNA by sequestering miR-34a, thereby modulating GFRA1 expression. Silencing circGFRA1 markedly reduces cell proliferation and triggers apoptosis in TNBC 40. CircRRM2 is upregulated in breast cancer tissues, where it acts as a ceRNA to sponge miR-31-5p/miR-27b-3p and activate IGF2BP1, leading to increased tumor cell invasion and migration 41.

CircCRIM1, which is secreted via adipocyte-derived exosomes, inhibits miR-503-5p and activates the OGA protein, thereby accelerating TNBC progression and metastasis 42. CircCD44 enhances cell proliferation, migration, and invasion by functioning as a molecular sponge for miR-502-5p. This interaction influences insulin-like growth factor 2 mRNA-binding protein 2 (IGF2BP2), resulting in changes in cell behavior 43, 44. Moreover, circBACH1 is upregulated in chemotherapy-induced breast cancer exosomes, increasing tumor cell invasion and drug resistance via the miR-217/G3BP2 axis 45.

Although CircFOXO3 is generally downregulated, its overexpression significantly suppresses TNBC cell proliferation and metastasis through the modulation of the WHSC1-H3K36me2-ZEB2 axis 46. CircRAD54L2 functions as an miR-888 family sponge, upregulating PDK1 expression, which facilitates breast cancer cell invasion and migration 47. circRREB1 directly interacts with GNB4 to activate the Erk1/2 signaling pathway, enhancing tumor cell migration and invasion 48. Notably, circCFL1 acts as a scaffold to increase HDAC1 binding to c-Myc, stabilizing the c-Myc protein and activating mutant TP53 expression. This process promotes TNBC stemness and immune evasion 49. CircTFF1 is markedly increased in breast cancer tissues, and its silencing inhibits the progression of breast cancer cells. circTFF1 acts as a molecular sponge for miR-326, facilitating breast cancer progression via the miR-326/TFF1 pathway. These findings identify circTFF1 as a novel oncogene in breast cancer 50.

### Circular RNAs that Promote the Distant Metastasis of Breast Cancer

**Figure 2:**
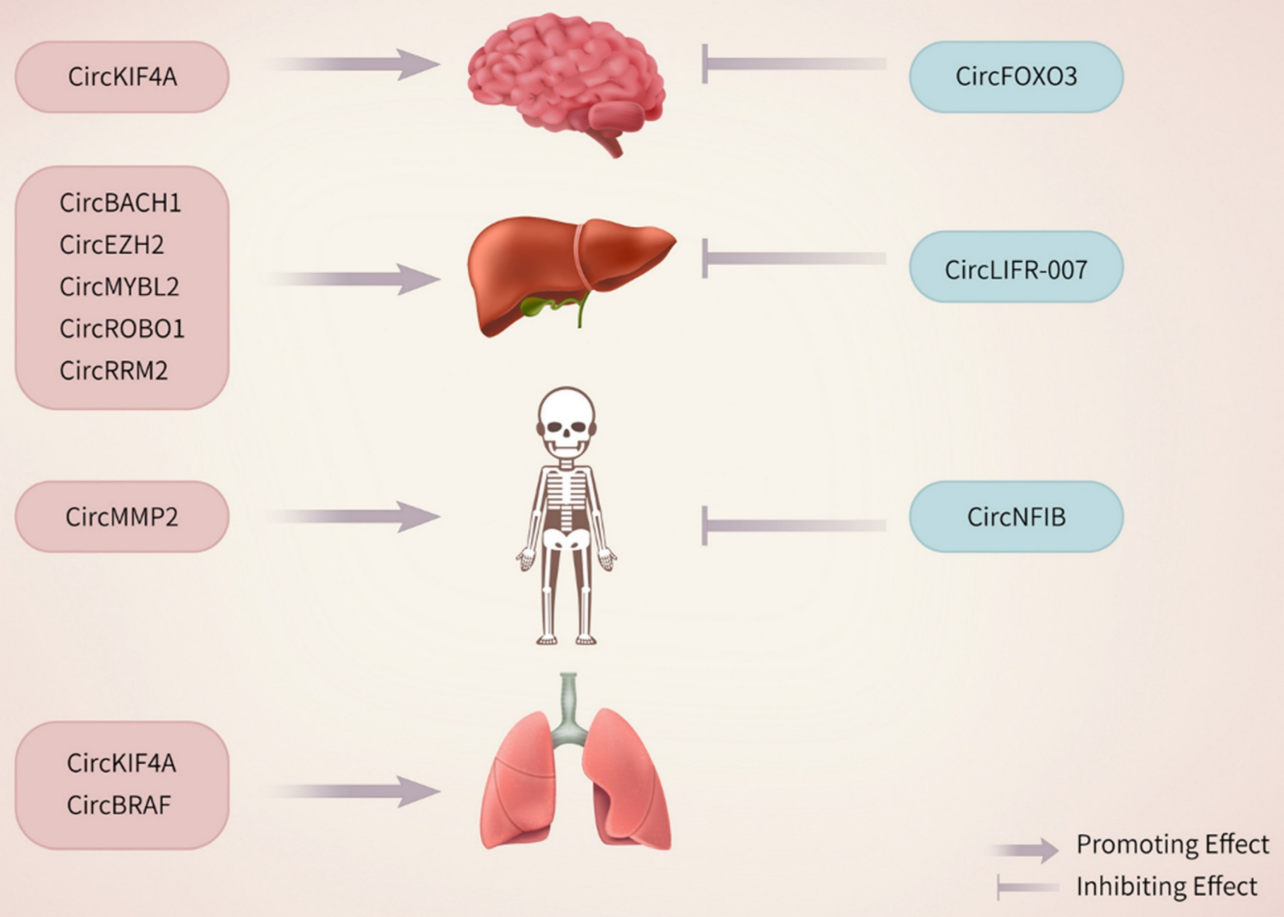
The stimulatory or inhibitory effects of differentially expressed circRNAs on metastasis.

The primary cause of death among breast cancer patients is distant metastasis. CircRNAs play pivotal roles in this process by regulating key molecular pathways, interacting with miRNAs, and modulating the tumor microenvironment. These processes facilitate the spread and growth of breast cancer cells in distant organs, including the brain, liver, bone, and lungs (Figure 2).

Research has shown that circKIF4A is upregulated in breast cancer tissues and is positively correlated with an advanced tumor stage and metastasis. Functionally, circKIF4A acts as a ceRNA, sponging miR-637 to prevent its suppression of STAT3 expression. STAT3, a well-known transcription factor, promotes the EMT, angiogenesis, and immune evasion, facilitating metastatic spread to distant organs 3. Both *in vitro* and *in vivo* studies have shown that silencing circKIF4A significantly reduces STAT3 activation, inhibits the expression of EMT markers such as N-cadherin and vimentin, and increases E-cadherin expression 51. The prometastatic roles of circKIF4A illustrates how circRNAs can influence key pathways to drive breast cancer metastasis. Targeting these circRNAs with antisense oligonucleotides (ASOs) or small-molecule inhibitors of the STAT3 and PI3K/AKT pathways is a promising approach for mitigating metastatic spread 52.

Besides promoting brain metastases, circRNAs also play a pro-oncogenic role in breast cancer liver metastases. The metastatic process involves a highly intricate, multistep cascade in which the EMT acts as a pivotal mechanism, promoting tumor cell detachment, migration, and invasion of distant sites 53. Zhang et al. showed that KLF5 is crucial for sustaining the EMT state and driving tumor progression in prostate cancer. This process is mediated by the upregulation of C-X-C chemokine receptor type 4 (CXCR4), which significantly promotes bone metastasis and enhances resistance to chemotherapy 54. High CXCL12 levels in organs such as the liver, bones, lungs, and lymph nodes activate the CXCR4/CXCL12 axis, promoting breast cancer progression and metastasis 55, 56. Moreover, the CXCR4‒CXCL12 axis, along with phosphorylated mTOR, can induce the EMT program in metastatic breast cancer 57. CircEZH2 promotes tumor cell proliferation and metastasis *in vitro* and *in vivo* by acting as a molecular sponge for miR-217-5p, thereby increasing Krüppel-like factor (KLF5) protein levels. KLF5 enhances FUsed in Sarcoma (FUS) transcription, facilitating the back-splicing required for circEZH2 formation. Furthermore, KLF5 activates CXCR4 transcription, triggering the EMT and driving the liver metastasis of breast cancer 34. Similarly, CircMYBL2 is overexpressed in both breast cancer cells and liver metastases. It facilitates the proliferation of breast cancer cells and their metastasis to the liver. CircMYBL2 promotes the EMT in breast cancer cells by sponging miR-1205, leading to the upregulation of the transcription factor E2F1, and by interacting with eukaryotic translation initiation factor 4A3 (eIF4A3) 58. Recent studies have indicated that CircROBO1 increases KLF5 expression by sequestering miR-217-5p. This upregulation of KLF5 increases the transcription of FUS, which in turn promotes the back-splicing of circROBO1. This process establishes a positive feedback loop involving circROBO1, KLF5, and FUS. circROBO1 promotes liver metastasis progression from breast cancer by upregulating KLF5, which suppresses selective autophagy mediated by afadin 35. CircRNAs significantly influence liver metastasis by regulating metabolic and signaling pathways. CircRRM2 is upregulated in metastatic breast cancer tissues and sponges miR-31-5p and miR-27b-3p, thereby activating IGF2BP1 and forming a positive feedback loop with MYC, which enhances cancer cell invasion and colonization in the liver 41. Furthermore, circCRIM1, which is secreted through adipocyte-derived exosomes, inhibits miR-503-5p and activates the OGA protein, facilitating TNBC liver metastasis 42.

CircRNAs also play a role in bone metastasis by affecting bone homeostasis. CircMMP2 interacts with β-catenin and PRMT5 to activate the transcription of bone remodeling factors such as S100A4 and LGALS3. These factors promote osteoclast activation and extracellular matrix degradation, creating a bone microenvironment conducive to cancer cell colonization. Targeting circMMP2 has shown efficacy in reducing the bone metastatic burden in preclinical models 59.

Moreover, circRNAs play critical roles in lung metastasis by modulating the EMT and angiogenesis. CircBRAF recruits KDM4B and IGF2BP3, regulating m6A RNA methylation and activating metastasis-related genes such as VCAN and MMP9. This process promotes cancer cell invasiveness and adaptation in the lung microenvironment, facilitating metastasis 60. The increased expression of these circRNAs in metastatic tissues indicates their potential as noninvasive biomarkers for assessing the metastatic risk and monitoring the treatment response.

Interestingly, while most circRNAs promote metastasis, some exhibit context-dependent roles. For example, circCFL1 stabilizes c-Myc by enhancing its interaction with HDAC1, promoting immune evasion and stemness in TNBC 49. Conversely, circNFIB suppresses metastasis by reducing arachidonic acid metabolism, which inhibits tumor growth and distant spread 61.

## Circular RNAs that Suppress Breast Cancer Cell Invasiveness

### Circular RNAs that Inhibit Breast Cancer Cell Invasion and Migration

The majority of circRNAs are known to promote breast cancer cell invasion and metastasis; however, certain circRNAs exhibit tumor-suppressive properties by inhibiting cell invasion, migration, and distant metastasis (Table 1). CircLIFR-007 suppresses breast cancer cell growth and spread *in vitro* and *in vivo*. Mechanistically, it promotes YAP phosphorylation by facilitating the cytoplasmic export of hnRNPA1, thereby enhancing the interaction between hnRNPA1 and YAP within the cytoplasm 59. circNFIB suppresses breast cancer cell proliferation and invasion by regulating phospholipase activity, which reduces arachidonic acid synthesis 61. CircDUSP1 functions as a molecular sponge for miR-761, increasing DACT2 expression and thereby inhibiting TNBC cell migration, invasion, and EMT. It increases cellular sensitivity to paclitaxel 62. circDUSP16 is significantly overexpressed in TNBC cells, tissues, and plasma-derived exosomes, and it promotes TNBC cell proliferation, migration, and invasion through the miR-1224-3p/TFDP2 axis. Silencing circDUSP16 suppresses tumor growth *in vivo*, highlighting its potential as a biomarker and therapeutic target for TNBC 63.

CircRPAP2 interacts with SRSF1, hindering its splicing regulation of PTK2 precursor mRNAs, leading to decreased PTK2 protein levels and subsequently inhibiting breast cancer cell migration and invasion 64.

CircKDM4B acts as a molecular sponge for miR-675, increasing NEDD4L expression. This process inhibits the PI3K/AKT signaling pathway and angiogenesis, significantly reducing breast cancer invasion and metastasis 65.

CircAHNAK1 expression is notably lower in TNBC tissues than in normal tissues. By sponging miR-421, it may suppress TNBC proliferation, migration, and invasion, increasing the expression of the tumor suppressor gene RASA1 66.

circFBXW7 acts as an miR-197-3p sponge and encodes the FBXW7-185aa protein, thereby inhibiting cell proliferation, migration, and tumor growth by upregulating FBXW7 expression in TNBC cell lines 67. circSEMA4B encodes the novel protein SEMA4B-211aa, which inhibits AKT phosphorylation, thereby blocking the PI3K/AKT pathway and significantly suppressing breast cancer cell proliferation and migration 68. Interestingly, although circAGFG1 generally has oncogenic properties in multiple cancers, its expression in breast cancer cells is associated with tumor-suppressive networks, where it regulates the EMT and key metastasis pathways 69.

CircRPPH1 functions as a molecular sponge for miR-542-3p, resulting in increased ARHGAP1 expression, which subsequently suppresses breast cancer cell proliferation, migration, and invasion 70. Additionally, *in vivo* experiments have demonstrated that the overexpression of circRPPH1 significantly suppresses tumor growth and metastasis in breast cancer.

CircNDST1 interacts with CSNK2A1 to suppress the PI3K-Akt signaling pathway and EMT, which in turn diminishes the invasiveness and metastatic potential of breast cancer cells 71. Studies have shown that circNDST1 expression is downregulated in breast cancer tissues and is associated with an improved patient prognosis.

In breast cancer cells, circPLK1 regulates the miR-1294/HMGA1 axis, resulting in reduced cancer cell migration, invasion, and tumor stemness, despite its usual oncogenic role in other cancers 72.

CircEIF3H interacts with HuR and IGF2BP2 to stabilize the HSPD1 and RBM8A mRNAs, thereby reducing the invasive characteristics of breast cancer cells 73.

These findings indicate that circRNAs influence breast cancer invasiveness and metastasis via crucial molecular pathways, positioning them as potential therapeutic targets and biomarkers for breast cancer diagnosis and prognosis.

### Circular RNAs that Inhibit Distant Breast Cancer Metastasis

Distant metastasis of breast cancer cells is a major cause of high mortality in patients. CircRNAs play essential roles in breast cancer metastasis by interacting with miRNAs, regulating gene expression, or modulating key signaling pathways, which can suppress distant metastasis (Figure 2).

CircFOXO3 is significantly downregulated in TNBC and acts as a potent suppressor of brain metastasis. It inhibits the nuclear localization of WHSC1, reducing H3K36me2 modifications and suppressing ZEB2 expression. This cascade halts the EMT, ultimately preventing TNBC cell invasion and brain metastasis. The low expression of circFOXO3 in patients correlates with a poor prognosis, highlighting its potential as a prognostic biomarker 46.

CircRNAs, including circLIFR-007, are essential for inhibiting liver metastasis. CircLIFR-007 promotes the nuclear export of hnRNPA1 and increases YAP phosphorylation, resulting in the downregulation of liver metastasis-associated proteins, including SREBF1 and SNAI1. This mechanism notably diminishes the liver metastatic potential of breast cancer cells, improving patient outcomes 74.

CircNFIB inhibits breast cancer metastasis by modulating arachidonic acid metabolism. A previous study showed that circNFIB sponges and suppresses the expression of enzymes that participate in the arachidonic acid metabolic pathway, such as COX-2 61. The inhibition of these enzymes results in a reduction in prostaglandin synthesis, which is essential for enhancing metastatic potential, with a primary effect on inducing inflammatory responses. Both *in vitro* and *in vivo* studies have shown that elevated circNFIB expression is associated with decreased invasion of tumor cells and reduced metastasis to bone, a frequent site of breast cancer spread. Clinical data further support its role, showing that circNFIB is downregulated in metastatic breast cancer tissues compared with primary tumors 61.

CircRNAs also suppress metastasis by targeting the EMT and angiogenesis pathways. CircKDM4B functions as a molecular sponge for miR-675, resulting in increased NEDD4L expression. This process inhibits the PI3K/AKT signaling pathway, reducing angiogenesis and cell migration. As a result, circKDM4B effectively reduces distant metastasis in TNBC models 65. circSEMA4B encodes the novel protein SEMA4B-211aa, which inhibits AKT phosphorylation, thereby blocking the PI3K/AKT pathway. This mechanism significantly suppresses breast cancer cell proliferation, migration, and distant spread 68.

Although circAGFG1 typically has oncogenic properties in other cancers, its expression in breast cancer is linked to tumor-suppressive networks. CircAGFG1 modulates key EMT- and metastasis-associated pathways, contributing to the inhibition of distant metastasis in specific breast cancer contexts 75.

## The Role of Circular RNAs in Angiogenesis During Breast Cancer Metastasis

Angiogenesis is essential for the distant metastasis of breast cancer. Tumors facilitate new blood vessel formation to secure the oxygen and nutrients essential for cell growth and migration 76.

CircRNAs that promote angiogenesis are critical for supporting metastatic progression by increasing vascularization within primary tumors and metastatic niches. CircRRM2 upregulates proangiogenic factors through the miR-31-5p/miR-27b-3p/IGF2BP1 axis, which enhances endothelial cell proliferation and vascular permeability, supporting the metastatic colonization of distant organs such as the liver. This mechanism is tightly linked to the aggressive nature of breast cancer metastasis 41. CircBACH1, which is secreted via chemotherapy-induced exosomes, facilitates angiogenesis by regulating the miR-217/G3BP2 signaling axis. By increasing vascular endothelial cell growth, circBACH1 promotes tumor vascularization and metastasis, particularly in drug-resistant breast cancer 45. CircKIF4A, which is overexpressed in TNBC, indirectly enhances angiogenesis through the activation of the STAT3 pathway, which upregulates key vascular endothelial growth factors and cytokines, enabling rapid tumor progression 3. These circRNAs act as essential modulators of angiogenesis, indicating their importance in establishing the vascular networks necessary for distant metastasis.

In contrast to proangiogenic circRNAs, a subset of circRNAs inhibits angiogenesis, counteracting metastatic progression. CircSEMA4B encodes a novel protein, SEMA4B-211aa, which inhibits angiogenesis by suppressing AKT phosphorylation and blocking the PI3K/AKT signaling pathway. This disruption of proangiogenic signaling pathways significantly reduces vascular formation and metastatic spread 68. CircNFIB reduces endothelial cell activation by downregulating arachidonic acid, a key metabolic pathway involved in angiogenesis. Its antiangiogenic properties inhibit tumor growth and metastasis in both *in vitro* and *in vivo* models 61. CircKDM4B functions as a molecular sponge for miR-675, leading to the upregulation of NEDD4L, which in turn inhibits angiogenesis by suppressing the PI3K/AKT pathway. This reduction in the angiogenic capacity is associated with decreased tumor cell migration and distant metastatic potential 65. These circRNAs highlight the potential of targeting angiogenesis as a therapeutic strategy to limit breast cancer metastasis.

CircRNAs regulate angiogenesis through multiple molecular pathways, demonstrating their versatile roles in shaping the tumor microenvironment. In the VEGF pathway, circRNAs such as circBACH1 and circKIF4A indirectly regulate the expression of VEGF, a central driver of angiogenesis. Targeting these circRNAs could help disrupt VEGF-mediated vascularization. Regarding m6A RNA methylation, CircRNAs such as circBRAF modulate epigenetic mechanisms such as m6A RNA methylation, which influence the expression of angiogenesis-related genes. This regulation highlights the role of circRNAs in the posttranscriptional control of proangiogenic factors 60. In PI3K/AKT signaling, both pro- and antiangiogenic circRNAs, such as circSEMA4B and circKDM4B, affect the PI3K/AKT pathway, underscoring their importance as therapeutic targets in circRNA-mediated angiogenesis. These mechanisms provide a molecular framework for understanding how circRNAs influence angiogenesis and metastatic progression 68.

The dual roles of circRNAs in angiogenesis highlight their potential as biomarkers and therapeutic targets in breast cancer metastasis. Proangiogenic circRNAs represent viable targets for the inhibition of vasculogenesis within metastatic neoplasms, whereas antiangiogenic circRNAs have potential for the derivation of novel therapeutic paradigms. For example, in the context of therapeutic targeting, the silencing of proangiogenic circRNAs, exemplified by circ_0008673^77^ or circHIPK3^78^, has the capacity to attenuate vascularization and curtail tumor dissemination.

Regarding biomarker applications, antiangiogenic circRNAs, such as circ_0047303, could function as reliable biomarkers for the evaluation of the angiogenic propensity and metastatic risk in breast cancer patients 79. In terms of combination therapy, circRNA-centered interventions may be integrated with extant antiangiogenic regimens, such as VEGF inhibitors, to augment therapeutic efficacy and overcome resistance. Advancements in delivery modalities, notably exosome-based circRNA carriers, portend the potential to surmount the challenges associated with circRNA stability and off-target consequences.

## The Role of Circular RNAs in Chemotherapy Resistance in Breast Cancer

The efficacy of breast cancer treatments is considerably hindered by chemotherapy resistance. The mechanisms of resistance involve increased expression of drug efflux proteins, increased DNA damage repair, and inhibition of apoptosis 9. Recent studies have identified circRNAs as key regulators of the development of chemotherapy resistance 80.

Research indicates that circRNAs play a role in the resistance of breast cancer to doxorubicin. The downregulation of circRNAs notably reduces drug resistance and inhibits the proliferation and metastasis of breast cancer cells, suggesting its potential as a therapeutic target 81. The suppression of circ_0085495 markedly decreases doxorubicin resistance and hinders the proliferation and spread of breast cancer cells 82. Furthermore, circRNA-CREIT is reportedly downregulated in TNBC cells that are resistant to doxorubicin. The therapeutic potential of circRNA-CREIT is further highlighted by the fact that its encapsulation in exosomes increases doxorubicin sensitivity in TNBC cells 16.

CircPVT1 is overexpressed in breast cancer tissues, driving tumor progression and drug resistance. It exerts this effect by stabilizing the ESR1 mRNA through competition for binding with miR-181a-2-3p, hence leading to an increase in the level of the ERα protein and subsequently activating target genes that are required for cancer cell growth and survival. Additionally, circPVT1 inhibits immune responses by binding to the MAVS protein and perturbing the RIG-I-MAVS complex, thereby inhibiting type I interferon signaling and enhancing immune evasion. Both mechanisms indicate a dual role for circPVT1 in tumorigenesis and therapy resistance. More importantly, therapeutic interventions with ASOs against circPVT1 resulted in the significant inhibition of proliferation and tumor growth, suggesting a new therapeutic intervention for ER-positive breast cancer 30.

In addition to modulating the chemotherapy response, circRNAs are implicated in shaping the tumor microenvironment, which contributes to both chemotherapy resistance and immunotherapy resistance. A study revealed that a high RM score, which is correlated with an immunosuppressive microenvironment, is a key factor contributing to the resistance to interventions such as PD-L1 blockade therapy. Within this regulatory framework, circWWC3 has been shown to upregulate IL-4 expression and subsequent cytokine secretion in breast cancer cells. This paracrine signaling subsequently induces PD-L1 expression in polarized M2 macrophages, thereby facilitating tumor immune evasion through PD-1/PD-L1 checkpoint activation. These findings highlight a potential link between circWWC3-mediated immunomodulation and circRNA-driven therapeutic resistance mechanisms in breast cancer 83.

Leveraging these insights, the integration of circRNA-targeted therapies with traditional chemotherapies or SG inhibitors such as ISRIB represents a promising approach for overcoming resistance and improving treatment outcomes in breast cancer patients.

## The Application of Circular RNAs as Biomarkers in Breast Cancer Patients

Advances in molecular biology technology have led scholars to recognize the significant potential of circRNAs in diagnosing, classifying, and assessing the prognosis of patients with breast cancer. The concept of circRNAs as biomarkers has progressed from early basic discovery to applied research 84, and some researchers believe that circRNAs show increased stability and conservation through their unique closed-loop structure 85. Others have proposed that circRNAs have specific tissue and temporal expression properties, making them important candidates as disease markers. In this work, the main discussion of circRNAs as biomarkers includes information on their feasibility as molecules with disease-specific expression patterns, molecular stability and the ability to be detected in body fluids 86.

The existing studies can be roughly divided into the following two main viewpoints according to different topics. In the first type of research, two representative views are discussed. CircRNA stability and specific expression in blood, urine, and tissue samples make it a potential noninvasive marker for breast cancer diagnosis 87. For example, elevated levels of circRHOT1 were consistently detected in circulating exosomes isolated from both BC patient sera and *in vitro* tumor cell cultures, establishing this circular RNA as a promising diagnostic biomarker for breast malignancies 88. In contrast, another view emphasizes the tissue-specific expression of circRNAs, such as the high expression pattern of circTP63 in estrogen receptor-positive breast cancer 89, which provides support for molecular typing. Thus, the study of circRNAs in breast cancer diagnosis reveals a basic consensus: their stability and specificity provide a new technical direction for early diagnosis. A discussion of how to overcome the limitation of detection sensitivity in the detection process is worthwhile, which is the focus of some research. A CRISPR-Cas13a/Cas12a-based system has been developed to enable simultaneous fluorescence detection of the breast cancer biomarkers circROBO1 and BRCA1^90^.

The second category of studies focuses on the potential of circRNAs as prognostic markers and is subdivided into two representative views. CircRNA expression levels are significantly linked to survival and the recurrence risk in breast cancer patients 84. For example, the downregulation of circCDR1as is associated with poorer outcomes in breast cancer patients. Another idea is that circRNAs may influence patient prognosis by regulating pathways associated with tumor metastasis 25. For example, circ PDSS1 promotes tumor metastasis by regulating the EMT, and its high expression is significantly associated with a poorer prognosis for patients with metastatic breast cancer 91. The circKIF4A-miR-375-KIF4A axis drives the progression of TNBC via the ceRNA mechanism. Thus, circKIF4A has potential as both a prognostic biomarker and a therapeutic target for TNBC 51. In addition, other studies have explained the role of circRNAs in the dynamic monitoring of breast cancer treatment response from the perspective of multiomics integration 25. The second view further expands the applications of circRNAs and forms a progressive relationship with the first view, namely, the overall application path from diagnosis to prognostic assessment.

Although circRNA research in breast cancer has advanced considerably, the clinical application of circRNAs remains challenging because of issues such as targeted delivery specificity and potential off-target effects 92. Future advancements in the integration of multiomics technologies with artificial intelligence are anticipated to expedite the translational use of circRNAs in the diagnosis and treatment of breast cancer 84, 93.

CircRNAs participate in breast cancer tumorigenesis and progression through various mechanisms, including acting as ceRNAs, encoding proteins, and regulating gene expression. Their inherent stability and tissue specificity further increase their potential as ideal biomarkers for breast cancer diagnosis, particularly in liquid biopsy applications. Moreover, targeting specific circRNAs holds promise as an effective therapeutic strategy, offering novel avenues for personalized breast cancer treatment 25.

The aforementioned studies provide valuable insights for applying circRNAs in breast cancer patients and advancing their clinical translation. Current research has largely overlooked certain aspects, particularly in terms of study design. A predominant focus on the singular role of circRNAs, such as in diagnosis or prognosis, has been noted, with an insufficient exploration of their potential as multifunctional markers. Second, regarding data analysis methods, most studies employ a single-omics analysis, with a notable deficiency in multiomics approaches to investigate the mechanisms of circRNAs. Third, from an argumentative point of view, the existing studies have focused more on the molecular biological functions of circRNAs and less on their feasibility and ethical challenges in clinical practice.

## Discussion

CircRNAs play dual roles in the distant metastasis of breast cancer, acting as both oncogenic drivers and tumor suppressors. This duality reflects the complexity of their biological functions and highlights the need for deeper mechanistic investigations to fully understand their context-dependent roles. CircRNAs influence multiple processes central to metastasis, including the EMT, angiogenesis, immune evasion, and metabolic adaptation. These processes enable breast cancer cells to survive, disseminate, and colonize distant organs, such as the brain, liver, bone, and lungs. Despite significant progress, several challenges remain in translating circRNA research into clinical applications.

One major challenge is the intricate regulatory networks in which circRNAs operate. The same circRNAs may exhibit opposing functions depending on the molecular context, such as interactions with specific miRNAs or RBPs. For example, circAGFG1, which is typically considered oncogenic in other cancers, has been shown to suppress metastasis in breast cancer by modulating EMT-related pathways. This finding highlights the importance of exploring the molecular environment and breast cancer subtype specificity when studying circRNA functions 75. CircRNAs play pivotal roles in regulating metastasis by targeting key signaling pathways, including PI3K/AKT, STAT3, and m6A RNA methylation, underscoring their central roles in metastasis regulation 3, 60, 94. However, the exact molecular mechanisms of these interactions are not fully understood, necessitating further research to explore these pathways at the single-cell and tissue-specific levels.

From a clinical perspective, circRNAs are promising diagnostic biomarkers and therapeutic targets in clinical settings because of their stability, tissue specificity, and role in modulating metastatic processes. For example, prometastatic circRNAs such as circKIF4A and circRRM2 may serve as predictive biomarkers for high-risk patients, whereas tumor-suppressive circRNAs such as circFOXO3 and circSEMA4B could be harnessed to develop novel therapeutic interventions 3, 41, 68, 95. Nonetheless, issues such as targeted delivery and off-target effects impede their clinical application. Delivery systems capable of specifically targeting circRNAs to metastatic niches, such as exosome-based carriers, need to be further developed and optimized. Additionally, understanding the role of circRNAs in therapy resistance, such as the contribution of circBACH1 to chemotherapy-induced angiogenesis, will be crucial for overcoming the current treatment limitations 45.

The effective delivery of circRNA-based therapeutics remains a significant challenge. Current delivery systems, including lipid nanoparticles and viral vectors, face limitations in specificity and efficiency, particularly in targeting metastatic or deep-seated tumors 18. For example, ensuring the stability of circRNAs in circulation and achieving precise tumor tissue targeting without affecting healthy tissues require advanced engineering of delivery platforms 96. Moreover, the lack of universal and scalable delivery strategies for encapsulating and transporting circRNAs hinders their widespread use in clinical settings 18. The biological effects of circRNAs often vary depending on the cellular and microenvironmental contexts. A circRNA that acts as a tumor suppressor in one cancer subtype might function as an oncogene in another, complicating the design of therapeutic strategies 97, 98. For example, circFOXO3 inhibits angiogenesis in breast cancer and may exert proapoptotic effects in other tissue contexts. Such functionality begs a context-dependent understanding of circRNA regulatory networks and demands caution when interventions are designed.

The possibility of off-target effects raises considerable safety concerns for circRNA-based therapies. RNA-based therapies, including circRNAs, can unintentionally interact with unintended molecular targets. Such interactions may lead to unpredictable side effects. For example, the widespread binding sites for microRNAs or proteins on circRNAs increase their likelihood of off-target interactions 99. Moreover, the immune system may recognize circRNA therapies as foreign, hence leading to inflammatory responses or autoimmune reactions 100. These safety concerns call for detailed preclinical assessments and the establishment of more precise mechanisms of targeting.

Another key area for future research is the integration of circRNA studies with multiomics technologies, such as single-cell RNA sequencing and spatial transcriptomics 101. These approaches can uncover the dynamic regulation of circRNAs within the tumor microenvironment, revealing their interactions with immune cells, endothelial cells, and stromal components, which may contribute to drug resistance in breast cancer chemotherapy, targeted therapy, and endocrine therapy 102. Such resistance poses significant challenges to cancer treatment, but the integration of advanced computational modeling and machine learning could aid in identifying novel circRNA signatures associated with metastatic progression, treatment outcomes, and potential strategies to overcome resistance 103.

In conclusion, while circRNAs provide valuable insights into the mechanisms of breast cancer metastasis, further research is needed to address the challenges associated with their dual roles and clinical applications. By focusing on the specific roles of circRNAs in different breast cancer subtypes and leveraging emerging technologies, researchers can unlock their full potential as biomarkers and therapeutic targets, paving the way for more effective strategies to combat breast cancer metastasis.

## Figures and Tables

**Table 1:** CircRNAs play dual roles in the invasion and migration of breast cancer cells

	Gene symbol	Location	CircBase ID (allas)	miRNA sponge	Target gene/pathway
Promote	CircKIF4A	chrX:69549254-69553539	hsa_circ_0007255	miR-637	circKIF4A/miR-637/STAT3
	CircEZH2	chr7:148529725-148544397	hsa_circ_0008324	miR-217-5p	FUS/circEZH2/KLF5/CXCR4
	CircMYBL2	chr20:42338602-42345122	hsa_circ_0060467	miR-1205	circMYBL2/miR-1205/E2F1
	CircROBO1	chr3:78763546-78796050	hsa_circ_0124696	miR-217-5p	circROBO1/KLF5/FUS
	CircXPO6	chr16:28112778-28113266	hsa_circ_0038773		c-Myc
	CircRAD18	chr3:8977554-8990254	hsa_circ_0002453	miR-208a/3164	circRAD18/miR-208a/3164-IGF1/FGF2
	CircEPSTI1	chr13:43528083- 43544806	hsa_circ_000479	miR-4753/miR-6809	circEPSTI1/miR-4753/6809-BCL11A
	CircPLK1	chr16:23691404-23701688	hsa_circ_0038632	miR‑1294	
	CircGFRA1	chr10:117,849,251-117,856,275,	hsa_circ_005239	miR-34a	miR-34a
	CircMMP2	chr16:55522454-55523736	hsa_circ_0039408		circMMP2(6,7)/β-catenin/PRMT5
	CircRRM2	chr2:10267001-10269281	hsa_circ_0052582	miR-31-5p/miR-27b-3p	IGF2BP1
	CircCRIM1	chr2:36764604-36764689	hsa_circ_0007408	miR-503-5p	miR-503-5p/O-GlcNAcase (OGA)/FBP1
	CircCD44	chr11:35226058-35227790	hsa_circ_0021735	miR-502-5p	CircCD44/miR-502-5p/KRAS
	CircBACH1	chr21:30698379-30702014	hsa_circ_0061395	miR-217	CircBACH1/miR-217/G3BP2
	CircFOXO3	chr6:108984657-108986092	hsa_circ_0006404		CircFOXO3/WHSC1-H3K36me2-ZEB2
	CircRAD54L2	chr3:51575513-51586079	hsa_circ_0001306	miR-888	CircRAD54L2/miR-888s/PDK1
	CircRREB1	chr6:7176887-7189555	hsa_circ_0001573		Erk1/2
	CircBRAF	chr7:140476711-140508795	hsa_circ_0007178		KDM4B and IGF2BP3
	CircCFL1	chr11:65622881-65623563	hsa_circ_0000328		CircCFL1 /HDAC1/c-Myc/mutp53
	CircTFF1	chr21:43782390-43786644	hsa_circ_0061825	miR-326	TFF1
Inhibit	CircLIFR-007	chr5:38481587-38481740	hsa_circ_0129040		YAP
	CircNFIB	chr9:14146687-14179779	hsa_circ_0086376		AA
	CircDUSP1	chr5:172195092-172196135	hsa_circ_0075043	miR-761	circDUSP/miR-761/DACT2
	CircRPAP2	chr1:92798947-92846430	hsa_circ_0000091		circRPAP2/SRSF1/PTK2
	CircKDM4B	chr19:5047486-5082515	hsa_circ_0002926	miR-675	circKDM4B /miR-675/NEDD4L
	CircSEMA4B	chr15:90760670-90764997	hsa_circ_0000650	miR-330-3p	circSEMA4B /miR-330-3p/PI3K/AKT
	CircAGFG1	chr2:228356262-228389631	hsa_circ_0058514	miR-195-5p	circAGFG1/miR-195-5p/CCNE1
	CircRPPH1	chr14:20811305-20811534	hsa_circ_0000515	miR-542-3p	circRPPH1/miR-542-3p/ARHGAP1
	CircNDST1	chr5:149918789-149919826	hsa_circ_0006943		PI3K-Akt
	CircGLIS3	chr9:4117767-4,125,941	hsa_circ_0007368	miR-146b-3p	circGLIS3/miR-146b-3p/AIF1L
	CircPLK1	chr16:23691404-23701688	hsa_circ_0038632	miR-296-5p	circPLK1/miR-296-5p/PLK1
	CircAHNAK1	chr11:62297840-62298224	hsa_circ_0000320	miR-421	circAHNAK1 /miR-421/RASA1
	CircFBXW7	chr4:153332454-153333681	hsa_circ_0001451	miR-197-3p	circFBXW7/miR-197-3p/FBXW7-185aa
	CircEIF3H	chr8:117668094-117671219	hsa_circ_0005231		HSPD1/RBM8A/G3BP1
